# Supplementary material for: Sexual health knowledge and needs among young adults with congenital heart disease
Source: PLoS One. 2021 May 5;16(5):e0251155. doi: 10.1371/journal.pone.0251155 (PMC8099087; doi:10.1371/journal.pone.0251155)
Supplement: S1 Questionnaire — (DOCX) [file pone.0251155.s001.docx]

1. **General characteristics**
2. What is your age? ( ) years old
3. What is your gender? ① Male ② Female
4. What is your marital status? ① Single ② Married ③ Other ( )
5. If married, for how long have you been married? Around ( ) years
6. What is your religion? ① Christian ② Buddhist ③ Catholic ④ No religion

⑤ Other ( )

1. What is your monthly household income?
   - 1. < KRW 1 million ② KRW 1 to 2 million ③ KRW 2 to 3 million

④ KRW 3 to 4 million ⑤ KRW 4 to 5 million ⑥ KRW 5 million <

1. What is your highest education level?
   - 1. High school ② Attending/graduated from un undergraduate school
     2. Attending/graduated from a graduate school ④ Other
2. What is the diagnosis of your congenital heart disease?

________________________________

1. What is/was the procedure for your congenital heart disease? (Date and number of procedures done), Name of surgery/procedure ________________________________
2. Are you taking any medications?

① Yes (Number: _____________, Name of medication: _____________), ② No

1. What was the level of tolerance regarding sexual activities in your family when you were growing up?
   - 1. Highly tolerant ② Somewhat tolerant ③ Rarely tolerant ④ Highly intolerant
2. Have you ever received education regarding sex? ① Yes ② No
3. If yes, how many times have you received it?

School: ( ) times Work: ( ) times Others: ( ) times

1. If yes, what kind of contents were included? Check (V) all that apply.

Pregnancy and childbirth ( ), Contraception ( ), Structure and function of the reproductive organs ( ), Sexual harassment ( ), Gender identity ( ), Sex counseling ( ), Sex life ( ), Geriatric sex ( ), Disabilities and sex ( ), Chronic diseases and sex ( ), Other

___________________________________________________________________________

Among the contents of sex education that you have received so far, what were the main contents? (Choose from the 10 examples above)

___________________________________________________________________________

1. Have you ever had sexual experience?
   - 1. No experience of intercourse ② I have experienced intercourse
2. Do you have something related to sex that you always wanted to know?

___________________________________________________________________________

1. **Knowledge Related to Safe Sex Practices**

The following questions are about knowledge related to sexual health. Please read it and check if it's correct or wrong.

| Item | True | False |
| --- | --- | --- |
| 1. When using a condom, make sure to leave some space near its tip |  |  |
| 2. Sexual partners need to remove condoms by rolling them from their bases, post ejaculation, to prevent semen from leaking out |  |  |
| 3. The reuse of a condom is possible after a thorough washing |  |  |
| 4. Daily prevention pills are an effective method of contraception |  |  |
| 5. Two days is the lifespan of the male sperm inside a woman's body |  |  |
| 6. Pregnancy is an unlikely result of adolescents taking part in sexual intercourse for the first time |  |  |
| 7. Taking the penis out of the vagina right before ejaculation is a safe and reliable way of preventing pregnancy |  |  |
| 8. Having sexual intercourse with an individual under the age of 16 is against the law |  |  |
| 9. The withdrawal method removing the penis just before ejaculation is the best way of preventing STD's (sexually transmitted diseases) |  |  |
| 10. Once a person has taken their first contraceptive pill, they are immediately immune from getting pregnant |  |  |
| 11. Pregnancy can be avoided by regular vaginal douching |  |  |
| 12. The consumption of a "morning after pill" within seven days of intercourse can prevent pregnancy |  |  |
| 13. Avoiding sexual intercourse according to the menstrual cycle is an effective contraception method |  |  |
| 14. The safety period of not getting pregnant is seven (7) days after and seven (7) days before menstruation |  |  |
| 15. Sexual intercourse during menstruation does not result in pregnancy |  |  |

**Thank you for participating in the survey.**
