# Supplementary material for: Sexual health knowledge and needs among young adults with congenital heart disease
Source: PLoS One. 2021 May 5;16(5):e0251155. doi: 10.1371/journal.pone.0251155 (PMC8099087; doi:10.1371/journal.pone.0251155)
Supplement: Attachment — Submitted filename: response to editor(2021-03-20).docx [file pone.0251155.s002.docx]

March 20, 2021

Dear Reviewer,

Thank you for taking the time to review our manuscript. We have revised the manuscript according to your feedback and hope it is satisfactory.

Should you have any questions or comments, please do not hesitate to let us know.

Sincerely,

The authors

| **Reviewer’s comments** | **Author’s response** |
| --- | --- |
| Please ensure that your manuscript meets PLOS ONE's style requirements, including those for file naming. | We formatted the manuscript according to PLOS ONE's style requirements. |
| Please include additional information regarding the survey or questionnaire used in the study and ensure that you have provided sufficient details that others could replicate the analyses. For instance, if you developed a questionnaire as part of this study and it is not under a copyright more restrictive than CC-BY, please include a copy, in both the original language and English, as Supporting Information. | Below is the reference information regarding the surveys used in this study. We have uploaded surveys as Supporting Information. Also we attached a questionnaire translated into English for adult with congenital heart disease.  **Adult with congenital heart disease**  https://forms.gle/NsEzCQZQXbVWku5AA  **Control group**  https://docs.google.com/forms/d/1E_dwxo1Fhum42cx9YnRrKjfZ2Ep4_gGUuq52mx2gpzg/edit |
| Thank you for including your ethics statement: "We received IRB approval (IRB No. 2019-1504) of 'A' hospital in Seoul and obtained permission from all participants. "Please amend your current ethics statement to include the full name of the ethics committee/institutional review board(s) that approved your specific study. Once you have amended this/these statement(s) in the Methods section of the manuscript, please add the same text to the “Ethics Statement” field of the submission form (via “Edit Submission”). | We added the full name of the institutional review board that approved our study in the Methods section (Data collection and Ethical consideration) on pages 6.  “We received Asan Medical Center Institutional Review Board approval (IRB No. 2019-1504) and the research was conducted in accordance with the Declaration of Helsinki.” |
| We note that you have indicated that data from this study are available upon request. PLOS only allows data to be available upon request if there are legal or ethical restrictions on sharing data publicly. For information on unacceptable data access restrictions, please see http://journals.plos.org/plosone/s/data-availability#loc-unacceptable-data-access-restrictions.    In your revised cover letter, please address the following prompts:    a) If there are ethical or legal restrictions on sharing a de-identified data set, please explain them in detail (e.g., data contain potentially identifying or sensitive patient information) and who has imposed them (e.g., an ethics committee). Please also provide contact information for a data access committee, ethics committee, or other institutional body to which data requests may be sent.    b) If there are no restrictions, please upload the minimal anonymized data set necessary to replicate your study findings as either Supporting Information files or to a stable, public repository and provide us with the relevant URLs, DOIs, or accession numbers. Please see http://www.bmj.com/content/340/bmj.c181.long for guidelines on how to de-identify and prepare clinical data for publication. For a list of acceptable repositories, please see http://journals.plos.org/plosone/s/data-availability#loc-recommended-repositories.    We will update your Data Availability statement on your behalf to reflect the information you provide. | It is difficult to share study data as it contains sensitive and personally identifiable patient information. These restrictions on sharing a de-identified data set were imposed by an Institutional Review Board Data Access Committee at Asan Medical Center. Data requests may be sent to the Data Access Committee at [irb@amc.seoul.kr](mailto:irb@amc.seoul.kr) (phone: 82-2-3010-7166) or to [ymim@dankook.ac.kr](mailto:ymim@dankook.ac.kr), (phone: 82-10-3472-7610). |
| Please provide additional details regarding participant consent. In the ethics statement in the Methods and online submission information, please ensure that you have specified what type you obtained (for instance, written or verbal, and if verbal, how it was documented and witnessed). If your study included minors, state whether you obtained consent from parents or guardians. If the need for consent was waived by the ethics committee, please include this information. | We added additional details regarding participant consent to the Methods section, on page 6(line 127-134) and replicated below:  “The contents of the questionnaire were explained face-to-face by the researchers before participants submitted written informed consent. We explained, in detail, the purpose and method of the study to participants and that the subjects could withdraw their consent at any time during the study period. After providing subjects with sufficient time to decide whether to participate in the study, each subject submitted a written consent form. To maintain the privacy of participants and allow them to answer sensitive questions freely, the survey was completed using online survey tools in each participant’s location of their choosing.” |
| The participants in the intervention group and control group should receive similar knowledge on sexual health from school, why there is difference in sexual knowledge between the groups? The same generic sexual health questionnaire was adopted to measure knowledge in both groups. Why there is significant difference in the result? It would be better to explain such phenomenon clearly in the discussion part. | The higher the severity of the congenital heart disease, the lower the likelihood of participating in normal school life and education due to frequent surgeries and hospitalizations. In addition, the more severe the symptoms caused by heart disease, the lower a person’s interest in sex. Thus, it is anticipated that the level of knowledge regarding sex will be lower for the intervention group. A sexual knowledge questionnaire for patients with congenital heart disease was not available, so the difference between the intervention and control groups may seen by evaluating the questionnaire with the same questionnaire as the general public. This additional information has been added to the discussion section (page 15, line 231-238) and replicated below. |
| Is there any significant difference in demographic data between the intervention and control groups, e.g. age, family attitude towards sex, sexual experience, gender and marital status? Is the significant result in sexual knowledge between groups due to the difference in demographic data between groups? It would be clearer to have such data analysis and findings reporting. | In Korean culture, family attitude towards sex, sexual experience, gender, and marital status can be very sensitive questions, so we could not survey these topics with the control group recruited online. Therefore, only questions about gender, age, and gender knowledge were included for the control group. I think this is a limitation of this research, and I hope that future research will be carried out by adding this part. |
