# Supplementary material for: Sexual health knowledge and needs among young adults with congenital heart disease
Source: PLoS One. 2021 May 5;16(5):e0251155. doi: 10.1371/journal.pone.0251155 (PMC8099087; doi:10.1371/journal.pone.0251155)
Supplement: Attachment — Submitted filename: Response to editor(2021-04-14).docx [file pone.0251155.s003.docx]

April 14, 2021

Dear Reviewer,

Thank you for taking the time to review our manuscript. We have revised the manuscript according to your feedback and hope it is satisfactory.

Should you have any questions or comments, please do not hesitate to let us know.

Yours sincerely,

The authors

| **Reviewer’s comments** | **Author’s response** |
| --- | --- |
| 1. Although it is mentioned in the discussion part that unplanned pregnancies can have potentially fatal consequences for fetuses, mothers, or both, risk of pregnancy and induced abortion in people with CHD should be explicitly stated in the introduction to justify the significance of adequate sexual education for this group of people and the need to conduct this study to identify their sexual knowledge. | Thank you for highlighting this aspect.  I have added relevant text to the introduction. |
| 2. Suggest to add reliability and validity of KSSP on page 6. | The validity and reliability at the time of development of the original tool and the validity of the Korean version at the time of development are added. In addition, I considered the reliability of the binary questions through the KR-20 (Kuder-Richardson Formula 20), but the correct answer rate varied from 29% to 99%, I thought it was difficult to confirm a reliability index. |
| 3. Suggest to add "indication of grade 1 based on the NYHA Functional Classification" in line 162 on page 8. | I have added relevant content concerning the NYHA Functional Classification 1. |
| 4. Use the abbreviation "CHD" in line 236 on page 15 to replace congenital heart disease. | I have used the abbreviation "CHD" as per your comment. |
